# Diabetes and Wound Angiogenesis

**DOI:** 10.3390/ijms18071419

**Published:** 2017-07-03

**Authors:** Uzoagu A. Okonkwo, Luisa A. DiPietro

**Affiliations:** 1 Department of Microbiology and Immunology, University of Illinois at Chicago College of Medicine, Chicago, IL 60612, USA; uokonk2@uic.edu; 2 Center for Wound Healing and Tissue Regeneration, University of Illinois at Chicago College of Dentistry, Chicago, IL 60612, USA

**Keywords:** diabetes, wound healing, angiogenesis

## Abstract

Diabetes Mellitus Type II (DM2) is a growing international health concern with no end in sight. Complications of DM2 involve a myriad of comorbidities including the serious complications of poor wound healing, chronic ulceration, and resultant limb amputation. In skin wound healing, which has definite, orderly phases, diabetes leads to improper function at all stages. While the etiology of chronic, non-healing diabetic wounds is multi-faceted, the progression to a non-healing phenotype is closely linked to poor vascular networks. This review focuses on diabetic wound healing, paying special attention to the aberrations that have been described in the proliferative, remodeling, and maturation phases of wound angiogenesis. Additionally, this review considers therapeutics that may offer promise to better wound healing outcomes.

## 1. Introduction

Diabetes mellitus type II (DM2) is a metabolic disorder defined by hyperglycemia due to insulin resistance. DM2 has become a major global health epidemic with a particularly large incidence in the United States. As of 2010 the global prevalence of DM2 was estimated at 280 million people, with recent measures by the Centers for Disease Control and Prevention (CDC) in 2014 indicating that 86 million Americans are in the pre-diabetic state and 29.1 million have been diagnosed with the disease [1]. DM2 is associated with numerous co-morbidities, including, but not limited to, cardiovascular disease, stroke, chronic renal failure, peripheral neuropathy, and diabetic skin wounds or ulcerations [2]. Diabetic skin ulcerations present as painful sores with disintegration of dermal tissue including the epidermis, dermis, and in many cases, subcutaneous tissue [3]. In diabetes, chronic skin ulcerations are common on the lower extremities, particularly the foot. Diabetic foot ulcers (DFU) affect 15% of diabetic patients. Of those patients with DFUs, 14–24% subsequently experience a lower extremity amputation, with the mortality rate from amputation approaching 50–59% five-year post-amputation [3,4,5,6]. Studies of the pathology of diabetic foot ulceration have focused on microbial invasion, epithelial breakdown, and impaired immune function as some of the causative factors for the non-healing phenotype [7]. One underlying factor that accompanies all diabetic ulcerations is poor vascular flow, a circumstance that impedes proper wound healing. Numerous studies have highlighted the importance of adequate vascular sufficiency and vessel proliferation in tissue repair and the lack thereof in diabetic wound healing [8]. More studies, albeit limited, have looked at whether disarrayed capillary remodeling and maturation of vessels might play a role in impaired diabetic wound healing. This review will synthesize the current findings in the literature about the role of appropriate capillary growth, function, and maturation in the context of diabetic wound healing.

## 2. The Anatomy and Maintenance of Blood Vessels

The human body is composed of a vast network of blood vessels that receive nutrient-rich, oxygenated blood from the heart starting with the largest in size called arteries, then subsequently decreasing in diameter to the arteriole, and finally to the capillary, where actual gas, nutrient, and waste exchange between vessels and tissue occurs. Waste is then carried back to the great veins of the venous system through venules. Capillaries, the cornerstone of nutrient diffusion, are so abundant in the body that no cell is more than 100–200 microns from one [9]. The anatomy of a capillary can be described as a microvessel that is composed of a one-cell layer thick lumen of endothelium with a diameter of 8–20 microns, just wide enough for the flow of erythrocytes and leukocytes [10]. The endothelial cells (EC) that constitute capillaries are dynamic cell types that have only recently been appreciated for their many activities. Endothelial cells line the lumen of every blood vessel in the body and are involved in filtration, hemostasis, barrier function, inflammation, and angiogenesis [11]. In the unwounded state, endothelial cells lining vessels are in a setting of quiescence demonstrated by the mostly basal expression of pro or anti-angiogenic stimuli in the vascular bed [12].

At the level of the capillaries, endothelial cells are in an intimate relationship with an enigmatic cell type called the pericyte. The pericyte is a type of mural cell embedded in the vascular basement membrane that wraps around endothelial cells [13,14]. Much remains to be discovered about this cell. Studies suggest that pericytes participate in regulation of capillary blood flow, clearance of cellular debris, stabilization, and paracrine communication of endothelial cells [15,16,17]. Loss of pericytes perturbs capillary function and results in extravascular leakage and edema [18]. The clear-cut identification of pericytes has long been a source of consternation. Due to their morphological similarities to vascular smooth muscle, the detection of distinct and ubiquitous markers that identify the unique pericyte population in vessels has proved to be an arduous task. Markers used with some success include α-smooth muscle actin (α-SMA) neural/glial antigen-2 (NG-2), desmin, Regulator of G-protein signaling 5 (RGS5), and platelet-derived growth factor receptor (PDGFR)-β [15,19]. Unfortunately, none of these markers are truly specific for the pericyte, and each marker is known to be expressed in other cell types. Nonetheless, several studies have linked some of these markers with pericyte function. Of note, PDGFR-β has been shown to be responsible for the recruitment of pericytes, and inhibition of PDGFR-β by imantinib results in decreased pericyte recruitment and migration in vitro [20]. In a mouse model, imantinib treatment caused decreased levels of NG-2^+^ pericytes in dermal wound tissue [21]. The role of PDGFR-β derives from interaction with its ligand, PDGF-β. PDGF-β deficient embryos show insufficient mural cell recruitment, resulting in uncontrolled EC development, vessel enlargement, permeability, and impaired perfusion [22,23]. Other factors that have been implicated in the maintenance of pericytes include the endothelial cell receptor, angiopoietin-2 receptor (Tie2) and its ligand, angiopoietin-1 (Ang1). Many studies showcase the importance of the Tie2/Ang1 complex to vessel maturity and integrity, including down regulation of the pro-angiogenic factor vascular endothelial growth factor (VEGF), and in turn, prevention of vascular leakage [24,25].

## 3. Vasculogenesis and Angiogenesis

Vasculogenesis is defined as the formation of new vessels from precursor cells that coalesce into primitive vascular networks. This process, which is prominent during development, contrasts with angiogenesis, which is the formation of new capillaries from established vasculature [26]. Angiogenesis occurs primarily during embryonic and post-natal development and is quite rare in the healthy adult state except in the female reproductive system [27]. Several female reproductive organs, including the ovary, endometrial lining of the uterus, and placenta undergo angiogenesis as part of their normal physiologic responses [28]. Both physiologic and pathologic angiogenesis are regulated by a balance of proangiogenic factors and anti-angiogenic factors. Vessels produced during non-pathologic angiogenesis are characterized by their refinement, integrity, and ability to deliver nutrients to tissues in a controlled, timely manner [29]. Disarrayed, runaway angiogenesis is a distinguishing feature of many pathologic processes including solid tumor growth and fibrosis [29,30]. In such pathological disease states, angiogenesis can be persistent, disorganized, and never reaches attenuation [31,32]. While basal levels of pro-angiogenic factors and the expression of their corresponding receptors are maintained during the healthy state of homeostasis, the diseased state exhibits upregulated expression of pro-angiogenic factors such as VEGF, fibroblast growth factor (FGF), and others to promote unfettered growth of vessel beds and continual proliferation and migration of endothelial cells [33]. Pathological angiogenesis is most often associated with malignancy, where excessive angiogenesis can continue without order or end as tumor growth [34]. Dysfunctional angiogenesis is also seen in Crohn’s disease, psoriasis, endometriosis, and rheumatoid arthritis [35]. Dysfunctional angiogenesis has been implicated in the pathophysiology of atherosclerosis and diabetic retinopathy as well as many other comorbidities that affect diabetic patients [36].

## 4. Angiogenesis in Wound Healing

**Figure 1:**
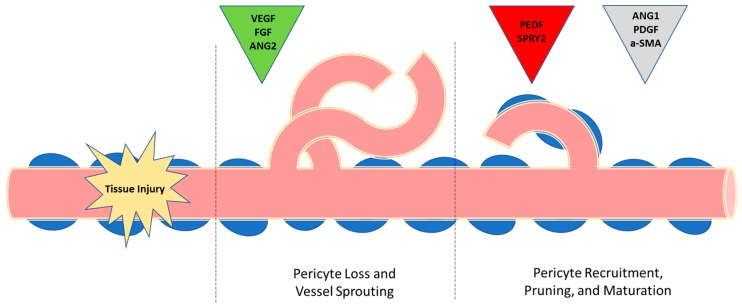
Events in wound angiogenesis. In the normal quiescent state, capillaries (pink) are surrounded by pericytes (blue). Following injury, the hypoxia that is created by the disruption of the vasculature stimulates the production of pro-angiogenic factors (green triangle), resulting in the sprouting of immature and disorganized new capillaries. In the remodeling phase, anti-angiogenic factors (red triangle) cause most of the newly formed capillaries to undergo apoptosis, and the capillary bed is pruned. Maturation factors (gray triangle) support the recruitment of stabilizing pericytes and the maturation of the basement membrane on the new capillaries. The result is a stable, well perfused capillary bed with a vessel density similar to normal uninjured tissue.

Wound healing is a complex process that can be divided into a series of stages that include hemostasis, inflammation, proliferation, and remodeling [37]. Prior to injury, the vasculature is in a state of quiescence in which blood vessels are adequately perfused to deliver sufficient nutrients, and oxygen to the tissue. Basal levels of pro-angiogenic factors such as VEGF and FGF in addition to anti-angiogenic factors such as Ang-1 and pigment epithelium derived factor (PEDF) are expressed to maintain a functional vascular network that is neither proliferating nor diminishing [38,39]. When an assault to tissue occurs that produces injury, this homeostasis is interrupted, leading to a hypoxic state. Hypoxia is an important activator of the endothelial cells in the injured and adjacent vasculature [40]. In this hypoxic environment, the innate immune system recruits leukocytes to the site of injury, with neutrophils being the first responders in the acute phase of inflammation [41]. Following the influx of neutrophils comes the later arrival of macrophages, and shortly thereafter the tissue reaches the zenith of the proliferative phase of wound healing. Here macrophages, emerging new capillaries, and loose connective tissue, characterized by edema and immaturity, form granulation tissue [42]. One hallmark of the proliferative phase of wound healing is robust angiogenesis. Following the oxygen gradient that was established by injury, numerous proangiogenic factors are produced in wounds. These factors, the most notable of which is VEGF, stimulate capillaries to form nascent immature loops and branches (Figure 1). VEGF has been shown to be one of the most important angiogenic factors in wounds, and its production lies downstream of hypoxia. Hypoxia following injury activates hypoxia-inducible factor-1 (HIF-1), a transcriptional activator that promotes angiogenesis by upregulating target genes such VEGF-A [43]. VEGF-A, the main isoform in the wound, binds to its receptors on endothelial cells, directing vessel growth [44]. VEGF and other pro-angiogenic factors guide vascular growth to areas of low oxygen starting from the wound periphery into the wound bed [40,45].

One of the defining attributes of wound angiogenesis is the creation of a disorganized and poorly perfused vasculature, characterized by a malformed capillary bed with blind-ended sprouts and torturous loops [29]. Although malformed, the amount of capillaries in wounds reaches numbers much higher than normal skin and peaks at approximately day 7–10 post wounding [46,47]. Following the apex of angiogenesis, a switch from pro- to anti-angiogenic factors is ushered in, and vessels begin to regress with the help of programmed cell death, termed apoptosis (Figure 1) [48,49]. The proliferative phase, which can be characterized as chaotic, robust, and abundant, is contrasted by maturation, a process that refines and selects for competent nascent vessels to become durable mature vessels similar to the pre-injured state [50]. As capillary refinement occurs, vessel stabilization is mediated by smooth muscle cell recruitment in the form of pericytes [51]. Pericytes are important to the stabilization and maturation of newly formed vascular bed. In wounds, pericytes are actively recruited in response to several factors, with the best described being PDGF [52,53].

Once pericytes arrive in the healing wound, they interact with endothelial cells and the basement membrane (Figure 1). Pericyte-covered capillaries in the wound bed are resistant to the anti-angiogenic factors which are produced during the remodeling phase of repair. Capillary pruning in wounds is mediated by the active production of several anti-angiogenic, vascular maturation factors, with the best described including pigment epithelium derived factor (PEDF) and sprouty-2 (SPRY2) [54,55]. PEDF is a member of the serine protease inhibitor (SERPIN) family, and is one the most potent anti-angiogenic factors in the vasculature [56]. It is constitutively expressed in unwounded skin and serves a powerful homeostatic factor. PEDF has been shown to induce EC apoptosis and to reduce the permeability of leakage-prone neovasculature [57]. A role for PEDF has been described in several other tissue pathologies with dysregulated angiogenesis, with one of the most widely studied being cancer. In a study by Chen et al., a popular drug used for the treatment of diabetes, metformin, was shown to decrease prostate cancer cell proliferation, migration and tumor growth through a mechanism that involved the upregulation of PEDF [58]. PEDF anti-angiogenic activity has been shown to reduce tumor growth and vascularity, and PEDF is currently under investigation as an anti-tumor and anti-angiogenic therapy [59]. In the context of skin wound healing, studies by our laboratory have shown that the production of PEDF is essential to vascular remodeling and maturation [60,61]. Sprouty-2 (SPRY2) is a second factor that has been demonstrated to assist in capillary remodeling in wounds. SPRY2 is an intracellular protein that inhibits mitogen-activated protein kinase (MAPK) signaling, ultimately downregulating the effect of VEGF on EC proliferation in wounds [55,62].

In addition to PEDF and SPRY2, another group of factors known to influence the normal progression of wound capillary growth and remodeling are the angiopoietins 1 and 2, which work in concert with the Tie2 receptor [63]. Ang1 is a potent maturation factor that stabilizes pericytes and ECs in capillaries. Angiopoietin-2 (Ang2) has an antagonistic effect and destabilizes vessels [64]. Both angiopoietins compete for binding to Tie2 tyrosine kinase receptor [65]. Consequently, during the normal angiogenic process, high levels of Ang2 are seen during the proliferative and pro-angiogenic phase of wound healing, while increased levels of Ang1 are seen during the maturation phase [66].

As the remodeling phase of wound healing ends, the tissue achieves normal vascular permeability, blood flow, and shows normal vascular branching [32]. Additionally, the high oxygen demand of the early stages of wound healing, a situation that activates many immune mediators and pro-angiogenic factors, returns to pre-injury levels [67]. The return to normoxia and normal oxygen demands signals a maintenance stage and return to quiescence. The quiescent environment includes anti-angiogenic mediators that ensure that vessel integrity is maintained and that endothelial cell migration, vessel sprouting, and branching are kept in check [68] (Figure 1).

In healing wounds, angiogenesis supports and intersects with the other ongoing proliferative activities and with the remodeling phase of repair [69]. During the proliferative phase, the new capillary growth in wounds is interwoven with multiple components of dermal repair. Fibroblast migration, proliferation, and collagen synthesis all occur during the same period as the angiogenic response. Similarly, epithelial proliferation and closure of the wound is also ongoing during the time of capillary growth. These many proliferative processes occur nearly simultaneously and support one another. For example, while new capillaries respond to the oxygen and nutrient needs of the proliferating tissues, stimulated epithelial cells produce VEGF to spur the capillary growth. During the remodeling phase, a phase triggered as oxygen levels return to normal, capillaries are pruned, the hyperproliferative epithelium thins to normal thickness, and the collagen in the wound bed undergoes maturation and cross-linking to achieve greater tissue strength. Recent studies suggest that capillary pruning and collagen maturation may interact in the remodeling phase, as the pruning of capillaries has been suggested to influence the final extracellular matrix (ECM) structure [70]. Like the proliferative phase, then, the remodeling phase exhibits a confluence of remodeling in capillaries, connective tissue, and epithelium.

## 5. Diabetes: An Altered Angiogenic State

As described above, angiogenesis in normal wound healing relies on a delicate balance between the promotion of vessel growth and proliferation and the promotion of vessel maturation and quiescence. The diabetic disease state can significantly perturb this balance, disrupting proper wound healing, tissue regeneration, and the restoration of a healthy vascular system. Perturbations in vascular integrity are also a feature of diabetes. Diabetic hyperglycemia, particularly in DM2, has been implicated in the progression of vascular disease in a multitude of both animal and clinical studies. The elevated systemic glucose levels seen in diabetic patients are the root cause of many micro and macrovascular complications that ultimately can affect angiogenesis [71]. ECs exposed to elevated blood glucose for extended periods of time have been shown to become dysfunctional, leading to integrity loss and increased susceptibility to apoptosis, detachment, and circulation into the bloodstream [72,73]. Free flowing, detached ECs have been shown to be a predictive marker of coronary heart disease and other pro-atherosclerotic processes in diabetes [74].

Insufficient angiogenesis plays a significant role in the pathogenesis of diabetic wound healing and micro and microvascular disease. Interestingly, though, while diabetic wounds have an angiogenic deficit, diabetes can lead to either increased or decreased angiogenesis depending upon the pathologic process. Numerous studies have shown that the diabetes-related changes in the angiogenic response can be tissue and/or organ dependent. For example, in diabetic retinopathy (DR) excessive angiogenesis occurs, leading to a pathology that is characterized by microaneurysms, hemorrhages, and vascular edema [75]. Along with increased capillary growth, a hallmark vascular modification seen in diabetic retinopathy is the loss of pericyte coverage in the retinal capillary network. This loss of pericytes is conducive to vascular edema and leakage. In response, the damaged capillaries experience hypoxia, leading to increased, abnormal expression of HIF-1 transcription factor and subsequent upregulation of VEGF-A, a known factor for increasing vascular permeability. This situation further contributes to the formation of an excessive neovasculature that is leaky and unrefined [75]. Importantly, an increase in the vitreous plasma levels of VEGF-A correlates with the severity of DR in diabetic patients [76]. Diabetic nephropathy (DN) is also characterized by excessive angiogenesis and resulting damage of the glomerular filtration system. The diabetic kidney may secrete excessive levels of VEGF-A in the initial stages of DN, which can lead to hyperpermeability of vessels, accelerated EC proliferation, and inhibition of apoptosis [77]. Studies by Cooper et al. showed in the early pathogenesis of diabetic nephropathy, mRNA levels of VEGF-A and its receptor VEGFR-2 are increased; in later disease progression levels of VEGF-A remain increased [78]. Disarrayed VEGF signaling is closely tied to impaired VEGF receptor activation, which is responsible for EC cell activation and proliferation, in addition to monocyte and endothelial progenitor cell (EPC) recruitment [75]. Abnormal receptor activation leads to increased circulating VEGF-A, due to decreased VEGF-A sensing in the vasculature, leading to such phenomena as plaque destabilization, and as mentioned previously, retinopathy [79].

## 6. Diabetes and Wound Angiogenesis

In contrast to diabetic nephropathy and retinopathy, diabetes leads to a decrease in angiogenesis in healing wounds. Diabetic wounds, impacted by insufficient angiogenesis, show decreased vascularity and capillary density [80]. Wound closure is greatly delayed in diabetes, and chronic non-healing wounds are common. An association of impaired angiogenesis to the pathologic wound repair seen in diabetic patients has been suggested by many studies. Below we review many of the described alterations in wound angiogenic response that are seen in the context of diabetes.

Macrophages, an important cell type of the innate immune system that are required for wound repair, have been shown to have altered functions in diabetic wounds [81]. In normal wounds, macrophages switch from a proinflammatory to pro-reparative phenotype, with the latter supporting tissue regrowth. In diabetic wounds, macrophage deficits include altered phenotypes that fail to stimulate tissue repair. One animal model of diabetes that has been widely used for wound healing studies is the db/db mouse. The db/db mouse is a genetic model of obesity, diabetes, and dyslipidemia that results from a mutation in the leptin receptor gene, and db/db mice are well documented to have significantly delayed healing [82]. In regard to macrophage function, Khanna et al. showed that macrophages at the wound site of db/db mice showed decreased efferocytosis, leading to increased apoptotic burden and inflammatory profile in the wound [83]. Since macrophages are an important source of VEGF and other pro-angiogenic mediators in wounds, the macrophage deficit may be linked to the documented decrease in wound angiogenesis that is seen in diabetic wounds. In one of the first studies to investigate the mechanisms that influence angiogenesis in diabetic skin wounds, Seitz et al. showed that VEGF-A protein and mRNA levels in wounds of db/db mice were significantly decreased compared to normal healthy controls [84]. Subsequent studies by Galiano et al. also identified this deficit, and went on to show that wounds of db/db mice treated with VEGF-A exhibited accelerated wound closure compared to untreated mice [85]. Of note, this study also showed that VEGF-A treated mice exhibited more of an early leaky, malformed vasculature and more edema until VEGF therapy was ceased [85].

In addition to the decrease in pro-angiogenic stimulus, several studies now demonstrate diabetes-associated changes in anti-angiogenic factors and capillary maturation factors in wounds. The production of the anti-angiogenic factor PEDF has been examined in the context of diabetic wound healing, although these studies investigated systemic serum levels rather than tissue expression levels. In one such study, PEDF was shown to occur at higher circulating levels in patients with diabetic foot ulcers compared to both non-diabetic and diabetic patients without DFU [86]. While this study might suggest that elevated levels of PEDF could negatively impact wound healing outcomes, the PEDF level was quantified only systemically and not within wound tissue.

One vascular maturation pathway that has been implicated in the deficits seen in diabetic wound angiogenesis is the Ang1/Ang2/Tie2 complex. In diabetic wounds it has been shown that the ratio of Ang1 to Ang2 is decreased, meaning that the ability of diabetic wound vasculature to progress to a mature phenotype is likely disturbed [87,88]. A role for Ang 1 as a maturation factor has also been described in streptozotocin (STZ)-induced diabetic mice. These studies have shown that topical application of neutralizing antibodies to Ang1 on wounded skin reduced the maturation of nascently formed blood vessels [89] Another study has shown that treatment of wounds of STZ-induced diabetic mice with transplanted bone marrow treated with Ang1 led to increased endothelial progenitor cells (EPCs) and increased neovascularization at day 7 post wounding [90].

MicroRNAs (miRNAs) are another class of molecule that can regulate angiogenesis and other aspects of wound repair, and miRNAs are known to be differentially expressed in the diabetic wound milieu. miRNAs are small, non-coding RNAs that are involved in post-translational modifications or gene silencing. Many miRNAs have been shown to be perturbed in diabetic wound healing, and specific miRNAs have been demonstrated to have modified expression in diabetic wound healing [91]. miR26-b is one miRNA that is highly expressed in diabetic ECs, and neutralization of this miRNA in diabetic wound models leads to increased wound closure and granulation tissue production [92]. Downregulation of miR-200b, shown to enhance TNF-α expression, leads to increased angiogenesis in diabetic wound skin [93]. In vivo and in vitro studies using local miR27-b, believed to affect levels of the anti-angiogenic molecule thrombospondin 1 (TSP1) in the wound bed, showed that restoration of miR27-b regulates angiogenesis in diabetic mouse models [94].

In the resolution and maturation phase of wound angiogenesis, platelet derived growth factor (PDGF) is one maturation factor that appears to be perturbed in the diabetic state. As mentioned above, PDGF encourages capillary maturation by nurturing and recruiting pericytes and retarding vessel regression [95]. PDGF has been extensively studied in diabetic skin wound healing, and db/db mice express lower levels of PDGF and its receptor in wounds [96]. Moreover, the topical application of PDGF has been shown to accelerate closure rates in diabetic wound healing [97]. PDGF is likely to have effects beyond capillary stabilization, as this factor is also a mitogen for fibroblasts. As further discussed below, recombinant PDGF is one of the only currently available growth factor therapies for non-healing diabetic ulcers.

In addition to changes in the production of pro-angiogenic and vascular maturation factors, the diabetic state leads to an inherently decreased population of endothelial progenitor cells (EPCs) from the bone marrow [98]. This deficit in turn, reduces the baseline vascularity in diabetic tissues and likely affects wound angiogenesis [99]. The EPCs of diabetics have a reduced capacity to produce functional angiogenic sprouts and tubes in ischemic models [100]. Without the appropriate function of these vital cells, processes such as granulation tissue formation, capillary growth, and collagen deposition are impaired in the wound bed [37,101]. Several studies suggest that EPC therapy might benefit diabetic wounds. For example, studies by Asai et al. showed that topical introduction of the sonic hedgehog (*Shh*) gene induced EPC proliferation, adhesion, and tube formation in vitro and increased wound vascularity in vivo in diabetic mice [102].

Given the many changes in pro-angiogenic and vascular maturation factors in diabetes, it is perhaps not surprising that the vascular architecture is known to be perturbed both in the normal skin and in the wounds of diabetics. Corrosion casting and scanning electron microscopy (SEM) studies have shown that diabetic patients suffering from diabetic microangiopathy in the toe region exhibit damaged capillary architecture and evidence of vascular leakage [103]. Deficiencies in the vascular architecture in diabetic wounds have also been noted. Recent studies in our laboratory have found that wounds of mice subjected to a high fat diet (HFD), a diet induced obesity model (DIO) that closely approximates DM2, present with more tortuous and aberrant architecture [104]. Overall, the diabetic state creates a large array of angiogenic deficits that occur during both the early and late stages of wound healing and affect both the proliferation and maturation of vessels. Many of the known anti-angiogenic and maturation factors in wounds have not yet been studied in the context of diabetes. In recent preliminary studies, though, we found that excisional wounds from db/db mice express lower levels of multiple anti-angiogenic and maturation factors during wound healing resolution as compared to control [105]. Conceptually, this suggests that vascular pruning and maturation may be delayed in diabetic wounds, a situation that might lead to chronic wounds or recurring wounds due to lack of a well-perfused and durable vascular bed. Table 1 gives a brief overview of the angiogenic events that are altered in diabetes. Further investigations of anti-angiogenic and maturation factor levels in the diabetic wound will need to be conducted to further evaluate the changes that occur in the resolution phase of healing.

## 7. Current Therapies to Ameliorate Diabetic Wound Healing

The hunt for effective therapies to treat the sizable patient population affected by the chronic, non-healing wounds brought on by diabetes has been elusive. While there has been research that has progressed from laboratory, to clinical trials, and finally to clinical practice, these treatments have failed to be the silver bullet that will heal chronic diabetic wounds. Here we discuss those available therapies that involve mechanisms that improve angiogenesis and vascular perfusion.

One therapy that has been heavily relied upon in the clinic has been hyperbaric oxygen therapy (HBOT). HBOT requires that a patient inhale 100 per cent oxygen in an enclosed chamber where the pressure has been increased to above that found at sea level. The treatment has been shown to improve tissue hypoxia, vessel perfusion, reduce inflammation and edema, and increase angiogenesis [106]. Numerous studies in patients with DFUs have shown that patients undergoing HBOT show increased healing rates and reduced risk of a major limb amputation [107,108]. Unfortunately, HBOT is cost-prohibitive to the average patient, and while it has an over 20-year track record in the clinic, is still not a complete answer for treating non-healing diabetic foot wounds. Another therapy that has been attempted is the use of growth factors such as VEGF and PDGF. As mentioned before, these factors are important in the proliferative and maturation phase of wound healing, respectively. Numerous animal studies have shown that topical application of VEGF and its isoforms improve wound healing in diabetic mice [85,109,110]. However, topical VEGF therapy in human DFUs with recombinant VEGF (rh-VEGF) (Telbermin) has been met with limited success. Although Phase I trials suggested that patients who received topical VEGF compared to placebo exhibited improved healing, the drug was abandoned after Phase II clinical trials demonstrated no significant effect [111]. PDGF treatment has also been investigated in both mouse model and clinical trials. Recombinant PDGF became available as becaplermin (Regranex) in 1997 for the treatment of DFUs [112,113,114]. Topical treatment with becaplermin in clinical trial showed a 43 per cent increase in wound closure versus placebo in patients, in addition to reduced time to wound close of 32 per cent and complete healing of ulcers in 57.5% of patients [112,113,114]. Unfortunately, becaplermin has been met with many issues. Firstly, it is an expensive treatment that may not be readily accessible or feasible for many patients. Additionally, adverse side effects of rash and burning sensation at the site of application, as well as increased risks of osteomyelitis and cellulitis have been reported [113]. The most worrisome adverse side effect from the drug is the possible increased risk of malignancy in users who undergo more than 3 tubes of topical treatment, warranting the FDA to release a black box warning for the drug in 2008 [111]. Consequently, topical growth factors, while having promising results in animal models, have not yet translated well to the clinic. While single growth factors have met with limited success in the treatment of wounds, the use of platelet derived therapies has been suggested as a possible improvement as it provides a myriad of factors [115]. Platelets themselves are a rich source of many growth factors, including PDGF, transforming growth factor β (TGFβ), FGF-2, epidermal growth factor (EGF), and VEGF. Platelet derivatives such as platelet-rich plasma, platelet gel, and platelet-rich fibrin therefore have been explored for repair and regenerative strategies for both hard and soft tissues. Some of the advantages of platelet derivatives include the polyfactor approach as well as the ability to prepare the derivatives from the patient’s own platelets, thus limiting patient exposure to exogenous agents. Beyond growth factors, multiple promising new therapies for diabetic wounds are currently under investigation. These include the application of cells such as stem cells and macrophages, and the use of sophisticated bioengineering approaches to provoke tissue repair responses.

## 8. Limitations of Current Knowledge and Future Directions

Our current knowledge of the pathology of DFUs and of the angiogenic response in diabetic wounds is incomplete. One major stumbling block to our understanding has been the lack of readily available animal models that sufficiently approximate human diabetic wound healing, especially chronic wounds. Moreover, many of the preclinical animal studies to date have focused on monotherapies, an approach which seems unlikely to be adequate for the treatment of the multifactorial problem of diabetic chronic skin wounds. Furthermore, work in diabetic wound healing has until recently focused only on the initial stages of wound healing and not the deficits that might prevent diabetic wound resolution. Adequate healing requires vascular maturation, including a return to quiescence and a normal vascular network. Much more work is needed to understand the differences during all stages of wound healing that occur in the diabetic state, with the goal of finding therapies and interventions to help drive these non-healing wounds to a state of health and integrity. Considering the enormous health care costs, decreased quality of life, and numerous comorbidities that are associated with diabetes, it is imperative that a holistic and multi-factorial approach to treating diabetes and to advancing wound care is sought.

## Figures and Tables

**Table 1:** Diabetes-associated changes in wound healing.

Event	Diabetes-Associated Changes	References
Normal Quiescent Capillary Bed	Microangiopathies, loss of pericytes	[80]
Proangiogenic Stimulus in Wounds	Decreased response to hypoxia, decreased production of pro-angiogenic factors, impaired receptor function, miRNA misregulation, macrophage dysfunction	[43,81,83,85,90,92]
Angiogenic Response in Wounds	Blunted, miRNA misregulation, decreased in endothelial progenitor cells	[91,93,94,101,102]
Capillary Pruning and Maturation during Wound Resolution	Not yet well studied, but altered production of anti-angiogenic factors reported	[17,86,89]

## Abbreviations

α-SMA	α-smooth muscle actin
Ang	angiopoietin
CDC	Centers for Disease Control and Prevention
db/db	genetically diabetic mouse model
DIO	diet-induced obesity
DN	diabetic nephropathy
DFU	diabetic foot ulcer
DM2	diabetes mellitus type II
EC	endothelial cell
ECM	extracellular matrix
EGF	epidermal growth factor
EPC	endothelial progenitor cell
FGF	fibroblast growth factor
HBOT	hyperbaric oxygen therapy
HFD	high fat diet
HIF	hypoxia inducible factor
MAPK	mitogen-activated protein kinase
NG2	neural/glial antigen-2
PDGF	platelet derived growth factor
PDGFR	platelet derived growth factor receptor
PEDF	pigment epithelium derived factor
RGS5	regulator of G-protein signaling 5
SERPIN	serine protease inhibitor
Shh	sonic hedgehog
SPRY2	sprouty homolog 2
STZ	streptozotocin
Tie2	angiopoietin-2 receptor
TGFβ	transforming growth factor β
TNF-α	tumor necrosis factor-α
VEGF	vascular endothelial growth factor
